# Draft Genome Sequence of *Pseudomonas* sp. Strain M7D1, Isolated from the Rhizosphere of Desert Bloom Plants

**DOI:** 10.1128/MRA.00441-19

**Published:** 2019-05-30

**Authors:** Matías Poblete-Morales, Nicolas Plaza, Romina Almasia, Gino Corsini, Evelyn Silva-Moreno

**Affiliations:** a Facultad de Ciencias de la Salud, Instituto de Ciencias Biomédicas, Universidad Autónoma de Chile, Santiago, Chile; b Departamento I+D, Regulaciones, Biopacific SpA, Ñuñoa, Santiago, Chile; c Instituto de Investigaciones Agropecuarias, INIA-La Platina, Santiago, Chile; Broad Institute of MIT and Harvard University

## Abstract

We announce the draft genome sequence of Pseudomonas sp. strain M7D1, isolated from the rhizosphere of a plant in the Atacama Desert bloom event. The genome sequence had 6,170,633 bp with a G+C content of 59.9%. This draft genome sequence gives information about the presence of genes related to iron acquisition, alleviation of abiotic stress, and other essential traits of plant growth-promoting rhizobacteria.

## Abstract (precis)

We announce the draft genome sequence of Pseudomonas sp. strain M7D1, isolated from the rhizosphere of a plant in the Atacama Desert bloom event. The genome sequence had 6,170,633 bp with a G+C content of 59.9%.

## ANNOUNCEMENT

The genus Pseudomonas represents rod-shaped and mobile aerobic bacteria that are ubiquitous residents of various terrestrial and aquatic environments and have an important ecological role (1, 2). Very few species are opportunistic pathogens of plants and animals. Most species are commensals, while others are beneficial for plants by taking part in their nitrification processes (2–4); they can also be used in decontamination (5, 6), among many other applications. Pseudomonas sp. strain M7D1 was isolated from rhizosphere samples of desert bloom plants from the Atacama region located in the north of Chile. For isolation of culturable bacterial, 1 g of roots with adhering soil was resuspended in 9 ml of a sterile saline solution (0.85% NaCl), and the mixture was homogenized by vortexing vigorously for 15 min. One milliliter of supernatant was serially diluted in a sterile saline solution (9 ml), and 100 μl of each dilution was inoculated in solid Burk’s N-free medium as a selection for nitrogen fixation (7). The agar plates were incubated at 25°C for 72 h before bacterial isolation in a new plate with Burk’s N-free medium. According to the morphological characteristics and biochemical profiles described previously (8), one isolate was classified as a Pseudomonas sp. belonging to the fluorescens group, while amplification and sequencing of the 16S rRNA (9) gene showed a shared identity of 97.5% with Pseudomonas granadensis LMG 27940^T^ and Pseudomonas kribbensis 46-2^T^, analyzed using the online tool EzBioCloud (https://www.ezbiocloud.net/).

The bacteria were grown on solid plates of LB medium (BD) at 22°C for 24 h, and one isolated colony was collected for extraction of the total genomic DNA using the NucleoSpin soil kit (Macherey-Nagel, Düren, Germany), according to the manufacturer’s protocols. Library synthesis was performed with the Kapa HyperPrep kit (Kapa Biosystems, Wilmington, MA, USA), and genomic sequencing on an Illumina MiSeq platform was performed by Omega Bioservices (Norcross, GA, USA), with 2 × 300-bp paired-end (PE) reads. A total of 5,066,576 reads were trimmed, normalized, and corrected with BBDuk BBNorm Error version 38.37 prior to *de novo* assembly using the Geneious Prime 2019.1 software. A total of 12 contigs were obtained (*L*_50_, 3; *N*_50_, 950,690 bp), with an average coverage depth of 85.5×. The length of the assembly is 6,170,633 bp, with a G+C content of 59.9%. Functional annotation was performed using the Rapid Annotations using Subsystems Technology (RAST) server 2.0 (10) (http://rast.nmpdr.org/rast.cgi) via RAST*tk* (11) and the NCBI Prokaryotic Genome Annotation Pipeline (PGAP), identifying a total of 5,655 coding sequences (CDS) and 73 RNAs. A comparison by average nucleotide identity (ANI) in EzBioCloud (12) with the species of the fluorescens group determined that the species with closest proximity are P. granadensis and P. kribbensis, with low identity values of 87.65% and 86.33%, respectively. A search was carried out using keywords for genes of interest (Table 1), as well as a verification of the genes in Artemis 16.0.0 (13).

**TABLE 1:** Traits of genes attributable to plant growth promotion in the genome of *Pseudomonas* sp. strain M7D1

Gene function	Gene, encoded protein, or other potential involvement in conferring PGP traits^*a*^	No. of contigs/start position–stop position/strand
Phosphate solubilization	Alkaline phosphatase	1/714119–719650/+
	Pyrroloquinoline-quinone synthase	4/76855–77607/+
	Exopolyphosphatase	4/381013–382515/+
	Polyphosphate kinase	4/384912–382687/−
	Phosphate transport ATP binding, *pstB*, *pstA*, *pstC*	4/533746–535527/−
	ABC transporter phosphate *pstS*	4/539031–538033/−
	Quinate/shikimate dehydrogenase	5/16647–14191/−
	Inorganic pyrophosphatase	8/225024–225551/+
Nitric metabolism	Uncharacterized subgroup of the nitrilase superfamily	1/176999–177859/+
	Nitrogen regulation protein NtrB	2/426503–427588/+
	Nitrogen regulation protein NR(I), GlnG (=NtrC)	2/427585–429021/+
	Flavohemoglobin/nitric oxide dioxygenase	3/130891–132072/+
	Anaerobic nitric oxide reductase regulator *norR*	3/130727–129165/−
	Dioxygenases related to 2-nitropropane dioxygenase	4/6562–7530/+
	Phosphocarrier protein kinase/phosphorylase, nitrogen regulation associated	4/304854–302575/−
	Plant-induced nitrilase transcriptional regulator in cluster	5/180525–179551/−
	Hydrolase, carbon-nitrogen family	6/278460–279641/+
	Nitrite reductase small subunit	7/36354–36037/−
	Nitrite reductase large subunit	7/38804–36351/−
	Isonitrile hydratase	7/107289–106603/−
Trehalose metabolism	Trehalose synthase	5/230432–227091/−
	Malto-oligosyltrehalose trehalohydrolase	5/208963–210765/+
	Malto-oligosyltrehalose synthase	5/212837–215611/+
Auxin biosynthesis	Auxin efflux carrier family protein	1/161599–162540/+
	Beta-chain tryptophan synthase	4/754233–755465/+
	Alpha-chain tryptophan synthase	4/755465–756274/+
Iron transport	Ferric iron ABC transporter (Fe^3+^)	3/257207–256098/−
	Iron siderophore receptor gene *fecA*	5/375215–372789/−
	Ferrous transporter (Fe^2+^), *efeU, efeO, efeB*	5/126350–130549 /+
	Siderophore biosynthesis nonribosomal peptide	7/116758–120159/+
	TonB-dependent membrane receptor	7/144607–142124/−
	Siderophore biosynthesis gene *pvdA*	7/158643–159980/+

a PGP, plant growth-promoting.

This draft genome report of *Pseudomonas* sp. M7D1, isolated from desert bloom plants, confirms the presence of genes related to the acquisition of inorganic compounds, the alleviation of abiotic stress in plants, and other essential characteristics of plant growth-promoting rhizobacteria (PGPR) (Table 1). It is important to note that this bacterium also possesses genes that encode molecules involved in the production of antimicrobial compounds, as well as those involved in the degradation and production of volatile compounds that can participate in the protection and promotion of plant growth. Future studies will be carried out to classify this new bacterial species and determine the potential *in vivo* effect of this PGPR.

### Data availability.

This whole-genome project for *Pseudomonas* sp. M7D1 has been deposited in GenBank under the accession number SSBS00000000. The version described in this paper is version SSBS01000000. The BioProject number is PRJNA531338, and the BioSample number is SAMN11356701.
